# Immediate Pose Recovery Method for Untracked Frames in Feature-Based SLAM

**DOI:** 10.3390/s24030835

**Published:** 2024-01-27

**Authors:** Hexuan Dou, Zhenhuan Wang, Changhong Wang, Xinyang Zhao

**Affiliations:** Space Control and Inertial Technology Research Center, School of Astronautics, Harbin Institute of Technology, Harbin 150001, China

**Keywords:** computer vision, visual SLAM, unmanned vehicles, localization, failure detection and recovery

## Abstract

In challenging environments, feature-based visual SLAM encounters frequent failures in frame tracking, introducing unknown poses to robotic applications. This paper introduces an immediate approach for recovering untracked camera poses. Through the retrieval of key information from elapsed untracked frames, lost poses are efficiently restored with a short time consumption. Taking account of reconstructed poses and map points during local optimizing, a denser local map is constructed around ambiguous frames to enhance the further SLAM procedure. The proposed method is implemented in a SLAM system, and monocular experiments are conducted on datasets. The experimental results demonstrate that our method can reconstruct the untracked frames in nearly real time, effectively complementing missing segments of the trajectory. Concurrently, the accuracy and robustness for subsequent tracking are improved through the integration of recovered poses and map points.

## 1. Introduction

Simultaneous localization and mapping (SLAM) is a technique that simultaneously estimates poses and maps the environment through perception. It holds significant relevance as a positioning method for autonomous unmanned vehicles, particularly in GNSS-denied scenarios. Cameras, serving as sensors for SLAM, offer the advantage of being lightweight and cost-effective. As a consequence, visual SLAM is extensively investigated and utilized.

Different from photometry-based direct methods for visual SLAM, feature-based methods extract image features from the environment and estimate poses through associated landmarks. Within many of these methods, features can be characterized with distinctive properties called descriptors, which can be recognized repeatedly. This attribute allows feature-based methods to outperform other methods in terms of place recognition and loop closure.

Similar to other approaches, feature-based SLAM encounters the challenge of robust perception for long-term autonomy [1], particularly in environments characterized by a large scale, low texture, or unmodeled dynamics. Failures in feature tracking are inevitable, leaving corresponding frames untracked and introducing unknown poses to the trajectory. Worse still, the absence of matchings for these features leads to a shortage of landmarks, posing a risk to the robustness and accuracy of tracking for subsequent frames.

In this paper, a method is introduced to immediately recover the lost poses, by which the robustness and accuracy of the subsequent SLAM procedure can also be enhanced. The contributions are as follows:

1. By analyzing the cause of tracking failures in feature-based SLAM, a method to reconstruct untracked frames is proposed that can recover the lost poses and map points, respectively;
2. A structure is designed in a SLAM pipeline to handle key information from past frames, expediting the reconstruction of untracked frames to achieve near-real-time performance;
3. By incorporating the recovered poses and map points into the optimization framework, a denser local map is maintained, contributing to a more accurate and robust SLAM procedure;
4. Based on open-source SLAM codes, the system with the proposed method is implemented, and experiments conducted on popular datasets validate the efficiency and efficacy of the approaches.

This paper is organized as follows. Related works on the avoidance of tracking failure and the response to tracking failure for feature-based SLAM systems are presented in Section 2. Section 3 analyzes the causes of introducing untracked frames, while investigating the feasibility and benefits of the reconstruction of these frames. The methodology of proposed pose recovery algorithms is presented in Section 4. The implementation details of software for experimental validation are introduced in Section 5. Experiments with the proposed method are conducted on datasets and presented in Section 6. Discussions on experimental results are provided in Section 7. The contribution of this paper is presented in Section 8, with a discussion of future works.

## 2. Related Works

### 2.1. Avoidance of Tracking Failure

Legacy feature-based SLAM systems, for instance, MONOSLAM [2] and FastSLAM 2.0 [3], are typically designed with static environment assumptions. In this context, inconsistent features are treated as sources of uncertainty in the state estimation process. These features are commonly regarded as disturbances or errors, whose adverse effects can be alleviated through filtering or optimization.

Nister D et al. [4] incorporated Random Sample Consensus (RANSAC) [5] into their visual odometry system in the motion estimation step. Since then, outlier rejection has gradually become essential in modern feature-based systems. Though inconsistent features are regarded as outliers to be eliminated in the front-end, tracking failure may occur in challenging environments. Under these circumstances, a large proportion of features exhibit inconsistencies with respect to static landmarks, rendering the task of outlier elimination challenging through statistical methods.

To address this issue, some feature-based methods are designed with an awareness of challenging environments to achieve robust SLAM procedures [6]. Techniques like semantic masking [7], dynamics modeling [8], and optical flow assistance [9] are implemented to identify nonstatic objects. These approaches are usually integrated into systems with image preprocessing, where features are expected to be extracted from static areas. However, the preprocessing incurs additional time, potentially impacting real-time performance. Besides, the presence of false positive masking may diminish the quantity of features, posing a risk to the precision and robustness of the algorithm.

On the other hand, some feature-based SLAM systems incorporate measurements from auxiliary sensors. To avoid tracking failure caused by the rapid reduction in the number of matched features with consistency, fusion with additional sensors is employed to enhance the confidence of SLAM results. For instance, with measurements from odometry [10] and inertial measurement units (IMUs) [11], ego-motion is introduced to improve pose estimation in feature-based SLAM. Moreover, with the use of depth cameras [12] and light detection and ranging (LiDAR) sensors [13], ranging perception can help compute the depth of features. Although additional sensor brings benefits to feature-based SLAM, purely visual methods should be further investigated to tackle the challenge of robust estimation and perception.

### 2.2. Response to Tracking Failure

If frame tracking encounters a failure, fail-safe measures should be implemented to prevent the interruption of SLAM software. Most state-of-the-art feature-based SLAM systems seek place recognition based on established landmarks to relocate the pose [14]. Some of the systems start a new SLAM session with reinitialization when tracking failure occurs [15]. Furthermore, within systems supporting multiple maps, such as ORB-SLAM3 [16], the initialization of a new map will be executed after a prolonged period of tracking failure. This reinitialization will create new landmarks, enhancing the tracking of subsequent frames and averting further tracking failure. Afterward, attempts are made to merge maps, aiming to achieve trajectories with global consistency.

Concerning the incomplete segments of a trajectory, some studies investigate the localization of untracked frames, typically employing offline approaches after processing the entire image sequence [17]. While these methods focus on promoting the completeness of trajectories, it is essential to note that offline algorithms may not directly contribute to improving online performance.

## 3. Untracked Frames and Reconstruction

Typical feature-based visual SLAM consists of the following stages:

1. Initialization: The computation of the relative pose between two or more frames is performed to triangulate an initial set of map points. In the condition of a stereo or depth camera, map points are initialized within one frame.
2. Front-end tracking: An incoming frame is tracked according to a feature matching relationship with tracked frames and/or modeled map points. New map points are modeled afterward.
3. Back-end optimization: By seeking loop closures and managing historical poses and map points, global optimizing is performed to suppress the drift and errors of a trajectory.

The localization of camera may fail due to the acceptance threshold set by the SLAM system for accuracy, and the corresponding frames therefore become untracked. Tracking failure may occur during monocular initialization and front-end tracking.

### 3.1. Tracking Failure during Monocular Initialization

Initial landmarks should be created before frame tracking, and such initialization in a monocular system shall be performed between two or more successive frames with parallax. Given matched feature points $\left\{p_{i}\right\}$ and $\left\{p_{i}^{\prime }\right\}$ in two temporal neighbor frames *F* and $F^{\prime }$, which are supposed to be projected from points $\left\{P_{i}\right\}$ in the real world, respectively, the rotation matrix R and transportation vector t of the camera center between the two poses satisfy

(1) $$p_{i}^{⊤}K^{-⊤}t^{\times }RK^{-1}p_{i}^{\prime }=0$$

according to epipolar geometry, supposing that the camera is calibrated with the intrinsic matrix K, and $t^{\times }$ is skew-symmetric of t. Defining the essential matrix

(2) $$E=t^{\times }R$$

and fundamental matrix

(3) $$F=K^{-⊤}EK^{-1},$$

F can be solved by the eight-point-algorithm [18]. Performing the Singular Value Decomposition (SVD) of E to calculate $t^{\times }$ and R, the extrinsic matrix

(4) C=[R|t]

between *F* and $F^{\prime }$ can be constructed, meaning the relative pose of the two frames is determined with a scale factor *s*. Then, the initial map points $\left\{P_{i}\right\}$ can be triangulated with C, indicating the initialization is complete. A RANSAC scheme is applied with more feature matches for a robust solution [19].

However, initialization may fail occasionally, especially in the following scenarios:

- Pure rotation: When the translation t is too small compared with rotation R, the numeric solution for E will be close to 0. To fulfill the initialization, the homography matrix
  (5) $$H=K^{-⊤}\left(R-\frac{tn^{⊤}}{d}\right)K^{-1}$$
  can be calculated by
  (6) $$pH=p^{\prime }$$
  with Direct Linear Transformation (DLT) and RANSAC if $\left\{P_{i}\right\}$ falls on the plane
  (7) $$n^{T}P+d=0.$$
  However, with t→0, the result of triangulation for $\left\{P_{i}\right\}$ will be unstable and even fail [20], leading to an initialization defect.
- During standby: As a part of the guidance, navigation, and control (GNC) module, the SLAM system on a robot commonly starts before movements for a mission. During the standby of a robot, for instance, in idle mode or the hovering of a drone, the view of the camera merely changes or vibrates randomly with a small amplitude. A reliable monocular initialization cannot be carried out with such low parallax until the steady motion of the camera occurs.
- Dynamic environment: If $\left\{P_{i}\right\}$ includes numerous points on moving objects, RANSAC may fail to obtain a consistent solution for E due to the violation of the epipolar constraints between rigid bodies with different velocities.
- Corrupt frames: When the view of the camera changes rapidly during initialization, distorted or blurred images might result in incorrect feature extraction and matching, leading to initialization failure. This would sometimes happen on a camera with a rolling shutter and an electrical synchronized global shutter. Furthermore, when in a low-textured environment, it is difficult to extract and match features properly, making it hard to finish the initialization.

**Figure 1:**
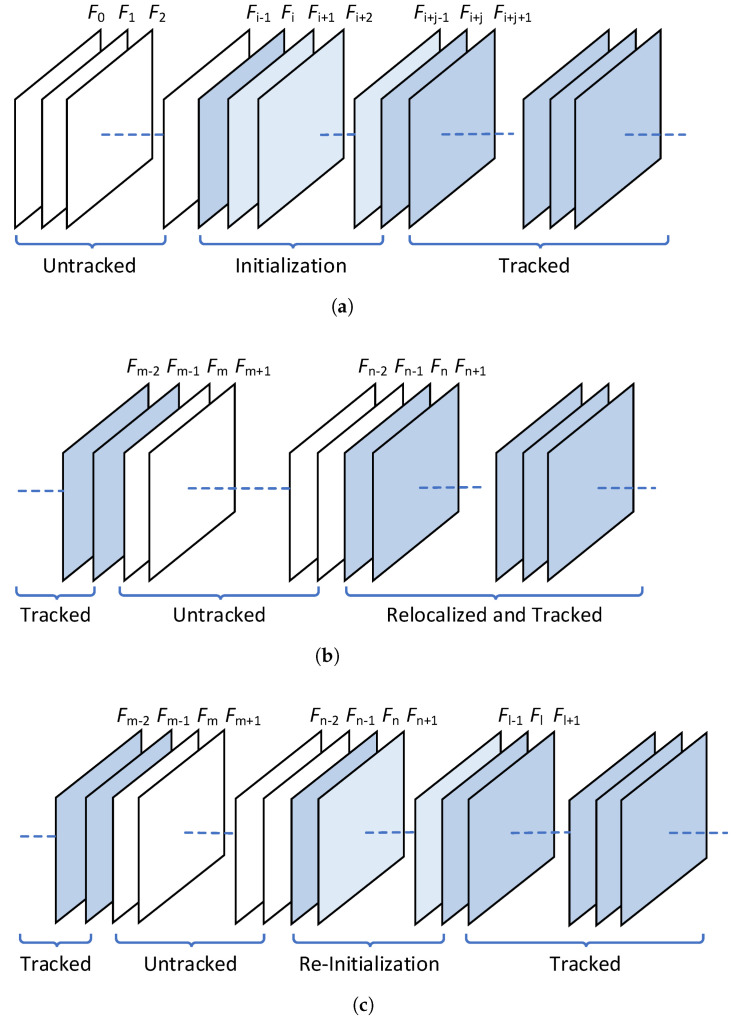
Illustration of untracked frames in different scenarios: (**a**) during initialization; (**b**) when tracking fails and performs relocalization; (**c**) when tracking fails and performs re-initialization. Frames in dark blue are successfully tracked by the SLAM system. Frames in light blue are skipped during initialization, whose pose can be estimated as well. Frames in white cannot be modeled within error tolerance and become untracked.

If no consistent F or H within error tolerance can be identified, or if there are too few reliable map points that can be triangulated, the monocular initialization between *F* and $F^{\prime }$ is deemed unacceptable by the SLAM system. An alternative initialization is sought between subsequent frames, denoted as $F^{\prime \prime }$ and $F^{\prime \prime \prime }$, while *F* and $F^{\prime }$ remain untracked consequently. This results in the introduction of unknown poses to the GNC system, as illustrated in Figure 1a.

### 3.2. Tracking Failure during Front-End Tracking

Once the system is initialized, with the matching between feature points $\left\{p_{i}\right\}$ on the incoming frame *F* and map points $\left\{P_{i}\right\}$, the pose of *F* satisfies

(8) $$s_{i}p_{i}=KTP_{i},$$

where $s_{i}$ is the scale factor and T=[R|t] is the transformation matrix. T can be solved by PnP, commonly in a bundle adjustment approach [21].

The tracking of *F* may fail as well in the following scenarios:

- Dynamic environment: If $\left\{P_{i}\right\}$ consists of numerous points on moving objects, which were considered stationary when mapped, the parts of these features turn to satisfying
  (9) $$\begin{array}{c} s_{i}p_{i}=KT\left(T_{r}P_{i}\right), \end{array}$$
  where $T_{r}$ is the transformation matrix of the corresponding moving object. These points would introduce additional errors to the PnP solver and lead to tracking defects.
- Textureless environment: If the camera enters a field of view containing massive textureless areas, such as glares or smooth walls where few image features can be extracted and tracked, there would not be a consist result of T due to insufficient matching between $\left\{p_{i}\right\}$ and $\left\{P_{i}\right\}$.
- Corrupt frames: Similar to the initialization, distorted, blurred, or low-featured images would result in incorrect feature extraction and matching, making the PnP solver unable to track the current frame correctly.

In the case where no pose estimation within the error tolerance can be found, the current frame becomes unmodeled. The SLAM system will then try to track the next frame continuously till an incoming frame can be tracked or relocalized correctly, leaving the frames untracked in the meantime, as shown in Figure 1b. In a SLAM system supporting multiple maps, a new initialization and map creation is to be performed if frame tracking fails for a long period, as shown in Figure 1c.

### 3.3. Feasibility of Reconstructing Untracked Frames

Although there are many sorts of reasons for a frame being untracked, a frame can be retracked and then reconstructed if it has adequately matched features with map points and if these matchings can result in a consistent pose estimation.

In the situation of initialization, though with correctly matched features, some frames are not suitable to create initial map points and are thus left untracked. Once initial map points are determined by the subsequent frames, these untracked frames could be tracked via proper feature matching, especially for the uninitialized frames caused by pure rotation, standby mode, and dynamic environments.

As for front-end tracking, though without sufficient matchings to the previous frame and map points, the untracked frames could match subsequent frames properly. Once the latter are modeled and new map points are created, the former could track these map points with the above matches and then calculate the poses. The untracked frames, particularly owing to the dynamic environment, could be reconstructed therefore.

### 3.4. Possibility of Improving SLAM Performance

Provided that some of the untracked frames could be reconstructed, the recovery of lost poses per se brings benefits to the GNC system. Furthermore, the complementary map points have the potential to improve the performance of the SLAM system.

**Figure 2:**
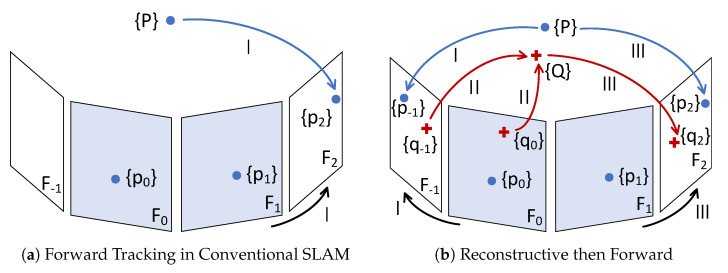
To estimate the pose of an incoming frame $F_{2}$ given tracked frames $\left\{F_{0},F_{1}\right\}$, (**a**) match features $\left\{p_{2}\right\}$ and map points {P} then solve PnP, or (**b**) I. estimate the pose of untracked frame $F_{-1}$ by $\left\{p_{-1}\right\}$ and {P}; II. create supplemental map points {Q} by $\left\{q_{-1}\right\}$ and $\left\{q_{0}\right\}$; III. match features $\left\{p_{2},q_{2}\right\}$ and map points {P,Q} then solve PnP. Note that $\left\{F_{i}\right\}$ would not be closely adjacent.

Compared with the conventional SLAM procedure, as shown in Figure 2a, more map points could be exploited when tracking incoming frames by reconstructing the untracked frames, as shown in Figure 2b. With map information enhanced by supplemental map points, the SLAM system is expected to achieve more accurate [22] and robust results.

### 3.5. Speeding-Up the Reconstruction Procedure

The untracked frames, some of which are able to be reconstructed, can be put into the SLAM pipeline with elapsed timestamps and treated as normal, newly incoming frames to be processed by the system. However, such processing will consume almost the same time as tracking normal frames, making it hard to recover the lost poses immediately and online.

A statistic on the time spending of ORB-SLAM3 processing the sequence *V2_02* of EuRoC datasets [23] is shown in Table 1. Whether successfully tracked or not, all image frames are to be processed by procedures, including ORB extracting, pose predicting, and part of local map tracking, and then determined whether to be accepted or not.

The execution of these operations for a single frame, even in the case of untracked ones, accounts for more than 65% of the total processing time of a typical frame. Nonetheless, it is important to note that features, matchings, and rough poses of the frame are generated meanwhile. It is of great convenience and benefit to manage these data, which are commonly deserted in most SLAM systems. These preprocessed data can be reused to reconstruct frames afterward, conserving time on redundant calculations.

## 4. Methodology

Compared with the typical, conventional forward monocular SLAM procedure, state estimation for untracked frames may be regarded as backward progress with similarities in principles and methods. However, as with complementary attempts with forward SLAM, the deployment of the recovery method should be taken care of due to the following two principles:

1. Accuracy: Rather than that of the recovery procedure, it is much more important to assure the accuracy of the forward SLAM process. The reuse and reconstruction of the untracked frames should never lower, if improve less, the accuracy of the main SLAM results, and offer additional posing information with precision in the meantime.
2. Efficiency: Though additional processing time cannot be averted during recovery, the immediacy of localization at the current time is of most significance to the SLAM system for robotic applications. The reconstruction of the poses and map features should consume excess computing time as little as possible in order to bring less latency to the main SLAM procedure for the best results.

Considering these principles, the proposed approach consists of three main components: the management of elapsed information, the recovery of lost poses, and the optimization of untracked frames.

### 4.1. Storage and Reuse of Information from Elapsed Frames

When processing the incoming frame, key information is buffered, including features, matching relationships, and rough pose. Once the current frame is considered untracked, the information is to be saved into the data structure. After a frame is considered to be retracked, these data will be exploited to reconstruct the intermediate frames, which ensures the efficiency of the pose recovery.

The outline of the management is presented in Algorithm 1. Features F are extracted in an incoming image frame *F*. F are then matched to features in neighbor frames or in the map, resulting in a matching relationship M together with the rough pose P of *F*. The accurate pose of *F* is then estimated and optimized with M, P, and the map points. If the pose of *F* within error tolerance cannot be determined, *F* is marked as untracked. In this circumstance, F, M, and P are saved to the data structure. These processed, associated data will speed up the reconstruction for *F* in the following steps.
**Algorithm 1** Storage of Information from Elapsed Frames**Input:** *Camera image***Output:** *Frame that is tracked or untracked with key information*1:Extract features F in the image and construct frame *F*;2:Match F to features in neighbor frames or in the map, resulting in a matching relationship M and rough pose P;3:Track *F* according to M and P;4:**if** *F* is tracked **then**5:   Model and save *F*, including pose and map points;6:**else**7:   Save *F* with F, M, and P to the data structure;8:**end if**9:**return** *F*.

### 4.2. Reconstruction of Untracked Frames

Once a frame $F_{0}$ is tracked after a series of untracked frames $\left\{F_{-i}\right\}$, the map points $\left\{P_{0}^{i}\right\}$ corresponding to $F_{0}$ can be projected to the adjacent untracked frame $F_{-1}$ according to Equation (8), if there are matched features $\left\{p_{0}^{i}\right\}$ and $\left\{p_{-1}^{i}\right\}$ between them. The transformation matrix $T_{-1}$ from $F_{0}$ to $F_{-1}$ can be recovered by minimizing the reprojection error as

(10) $$T_{-1}^{*}=\arg\min_{T_{-1}}\frac{1}{2}\sum_{i=1}^{n}{∥p_{-1}^{i}-\frac{1}{s_{i}}KT_{-1}P_{0}^{i}∥}_{2}^{2},$$

which is called a motion-only bundle adjustment.

After pose recovery, new map points $\left\{Q_{0}^{j}\right\}$ can be unprojected by additional matches $\left\{q_{0}^{j}\right\}$ and $\left\{q_{-1}^{j}\right\}$, which satisfy

(11) $$s_{m}^{j}q_{m}^{j}=KT_{m}Q_{0}^{j},m=-1,0.$$

A 4×4 linear homogeneous equation

(12) $$AQ_{0}^{j}=0$$

is then formulated and a numerical solution to homogeneous coordinates of $Q_{0}^{j}$ can be solved by SVD. Afterward, the map points $\left\{P_{-1}^{k}\right\}=\left\{P_{0}^{i},Q_{0}^{j}\right\}$ can be tracked by other unmodeled frames.

If there is a good recovery for $T_{-1}$, recursive operations can be executed to recover the poses for the rest untracked frames. In case there is not a good optimization result for $T_{-i}$ owing to frame corruption or other reasons, an estimation for the pose by epipolar constraint could be carried out, or the frame can just be skipped, and an interpolation for the pose can be performed later if necessary.

### 4.3. Local Optimization for Poses and Map Points

Modern feature-based SLAM systems perform key-frame-based local optimization with corresponding map points to improve the accuracy for positioning and mapping [14,24]. Similar optimization for recovered frames and map points can be conducted to reduce errors. Furthermore, by taking the recovered into the local mapping framework of further tracking, a more optimal result for incoming frames would be achieved with additional information.

The outline of reconstructing for untracked frames and local mapping, including the untracked and the incoming ones, is presented in Algorithm 2. For an untracked frame $F_{-i}$, which is adjacent to a tracked or retracked frame $F_{-(i-1)}$, an estimation for relative pose to $F_{-(i-1)}$ or its related key frame is performed exploiting preprocessed data $F_{-i}$, $P_{-i}$ and ${\left\{M_{j}\right\}}_{-i}$, if applicable, from the aforementioned data structure in Section 4.1. If the pose of $F_{-i}$ within error tolerance can be determined, $F_{-i}$ is marked as retracked, meaning that $F_{-i}$ is successfully reconstructed. Otherwise, $F_{-i}$ remains untracked and is skipped.

If the retracked frame $F_{-i}$ is novel in observing new map points, it will be constructed as a key frame. This key frame will undergo bundle adjustment with local key frames that observe covisible map points, and new map points will be constructed accordingly.
**Algorithm 2** Reconstruction and local mapping of the untracked frames**Input:** *Untracked frames $\left\{F_{-i}\right\}$ with preprocessed data and adjacent tracked frame $F_{0}$***Output:** *Poses and map points affiliated with $\left\{F_{-i}\right\}$*  1:**for** each i∈[1,n] **do**  2:   Estimate the pose of $\left\{F_{-i}\right\}$ with $F_{-(i-1)}$ or related key frame, exploiting $F_{-i}$, $P_{-i}$, and ${\left\{M_{j}\right\}}_{-i}$;  3:   **if** **not** Error below threshold **then**  4:     **continue**  5:   **else**  6:     Track and model $\left\{F_{-i}\right\}$  7:     **if** $\left\{F_{-i}\right\}$ is novel **then**  8:        Construct key frame from $\left\{F_{-i}\right\}$;  9:        **while not** Normal SLAM is local mapping **do**10:          Perform local mapping for the current key frame;11:          Construct and export new map points;12:        **end while**13:     **end if**14:   **end if**15:   Output the pose;16:**end for**17:**return** Poses for $\left\{F_{-i}\right\}$ and supplement map information

## 5. Implementation Details

To validate our methodology, a SLAM system is implemented based on ORB-SLAM3 codes. Subsequent experiments are conducted using this software implementation.

### 5.1. System Overview

In parallel with the original three threads: tracking, local mapping, and loop and map merging, a new thread, recovery, is introduced to manage untracked frames using the proposed approach. The recovery thread consists of two main components, a data storage module and a frame recovery module.

**Figure 3:**
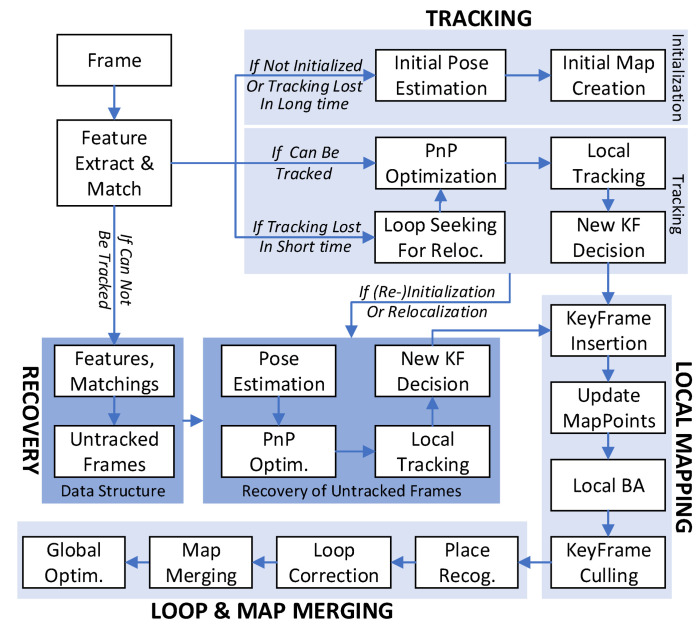
Main components of the proposed system. The parts in light blue are baseline ORB-SLAM3 threads, and the part in dark blue is the recovery thread.

The outline of implemented system is displayed in Figure 3. ORB features are extracted from the incoming image frame and matched with the last frame or its related key frame around feature pixels with the least hamming distance of descriptors. Pose estimation of the current frame is then performed in the tracking thread exploiting the matching relationship. If tracked, the frame will be determined to be a key frame according to novelty and passed into the local mapping thread for map point creation and local bundle adjustment. The loop and map merging thread is running for place recognition, loop closure, and map merging.

If the current frame cannot be tracked, the relevant data will be transferred to the recovery thread, where efforts will be made to retrack the frame. The recovery thread consists of a data storage module and a frame recovery module.

### 5.2. Data Storage Module

In this module, the elapsed untracked image frames with ORB features, prematched pairs, and roughly estimated poses are constructed and preserved, as stated in Section 4.1 and Algorithm 1. Pointers to these frames are stored in a stack. Once initialization, reinitialization, or relocalization is performed by the tracking thread, the stack will be passed to a new detached thread of the frame recovery module.

### 5.3. Frame Recovery Module

This module processes the untracked frames recursively in reverse order. For one untracked frame, a preliminary estimation of the relative pose is either loaded from storage or computed with the last tracked/retracked frame through matched features. Subsequently, PnP optimization is executed to enhance pose estimation according to Equation (10), and local mapping is performed with associated key frames and map points by bundle adjustment. Conversely, frames whose pose cannot be accurately estimated will be flagged as unrecovered, avoiding adverse impacts on subsequent recovery processes.

Through the above operations, the pose of this untracked frame is estimated, serving as a supplementary contribution to the missing segment of the trajectory. To further benefit the SLAM procedure, the retracked frames are selected and incorporated into the key frame database.

If a retracked frame contains a large number of new features or exceeds a specified time interval since the last key frame was generated, the frame is to be constructed into a key frame together with new landmarks. The key frames derived from retracked frames, along with conventional key frames, undergo a local bundle adjustment performed by the local mapping thread. This process enriches the key frame database and map point database, enhancing the subsequent tracking procedure.

### 5.4. Parameters Setting

To underscore the credibility of advantages conferred upon further tracking by recovered frames, the ORB-SLAM3 threads remain unchanged together with default parameters. The aforementioned manipulations of untracked frames in the frame recovery module are similar to that of the tracking thread during implementation, with minimal alterations to most hyperparameters. To maximize the utilization of recovered frames, only the thresholds for accepting feature matching are halved during recovery. This adjustment only affects the initial pose estimation of the retracked frames.

## 6. Experiments

### 6.1. Design of Experiments

Image sequences from datasets are processed by the aforementioned software, producing poses of key frames as trajectories and map points as maps. To prove the effectiveness of our method, the metrics of trajectory together with the completeness of the trajectory are compared between our method and ORB-SLAM3. The experiments were conducted on a computer with CPU *Intel Core i7-12700K@3.6GHz* and without the utilization of GPU acceleration.

#### 6.1.1. The Choice of Datasets

Our approach is valid in the presence of untracked frames. Consequently, the challenging sequences from popular datasets are selected for the experiments. When processing these sequences, conventional SLAM software is expected to experience initialization failure or tracking failure.

Some sequences in the visual SLAM datasets TUM [25] and EuRoC are suitable for the experiments, where pure rotation, corrupt images, moving objects, or textureless environments introduce untracked frames to the SLAM system. Namely, the ground robot sequences *pioneer_slam, pioneer_slam2, pioneer_slam3* and hand-held camera sequences *walking_xyz, walking_halfsphere, walking_rpy* in the TUM datasets are chosen in this experiment, together with the drone sequence *Vicon Room 2 02* in the EuRoC datasets.

#### 6.1.2. Evaluation Metrics

Absolute Trajectory Error (ATE) is used to measure the accuracy and consistency of the localization, and Relative Pose Error (RPE) is used to measure the drift of estimated poses. Root Mean Square Errors (RMSEs) of absolute trajectory and relative poses are calculated and compared to evaluate the accuracy of the SLAM system by convention.

To evaluate the completeness of the trajectory, an evaluation metric named Trajectory Completeness Rate (TCR) is defined in [15] as

(13) $$\eta =\frac{L_{est}}{L_{gt}}\times 100\%.$$

In this paper, $L_{gt}$ is the number of total frames, and $L_{est}$ is the number of successfully tracked frames, defined as $L_{est}=L_{gt}-L_{lost}$, where $L_{lost}$ is the number of frames whose poses cannot be determined. Our method is evaluated by online TCR, with $L_{est}$ counting the immediately determined frames only and overall TCR with $L_{est}$ including successfully recovered frames also.

To indicate the enrichment of the map, the total map points by frame are counted and compared. To evaluate the efficiency, the processing times of recovering the untracked frames in our method are recorded together with frame-tracking times, and the mean track times of all frames are calculated. The mean processing time for such operations is also provided for reference.

#### 6.1.3. Arrangements

To generalize the conclusion, experiments were conducted in monocular mode. A Sim(3) transformation was employed to align the monocular key frame trajectory with ground truth, utilizing the Umeyama Algorithm [26] for scale alignment. If multiple maps are created owing to a long tracking time failure, the trajectories are transformed and aligned to the origin, respectively, meaning that ATE and RPE will be underestimated in comparison with scenarios with fewer maps.

Untracked frames whose pose cannot be recovered within the acceptance threshold are marked as unrecovered, and they are counted in $L_{lost}$, calculating the overall TCR in our method. The poses of key frames constructed from recovered frames are recorded into the trajectory. Some of the output is processed and plotted by EVO [27], a trajectory evaluation tool for SLAM.

Since there is not a clear limitation of processing time for each frame in the circumstance of datasets, the strategy of key frame insertion by the recovery thread is set to unlimited in the experiments. This means that no attempt will be made to track the incoming frame until all the intermediate untracked frames are retracked and locally bundle-adjusted, which brings a slight halt but sharpened effect in accuracy and robustness for further tracking.

### 6.2. Experimental Results

**Figure 4:**
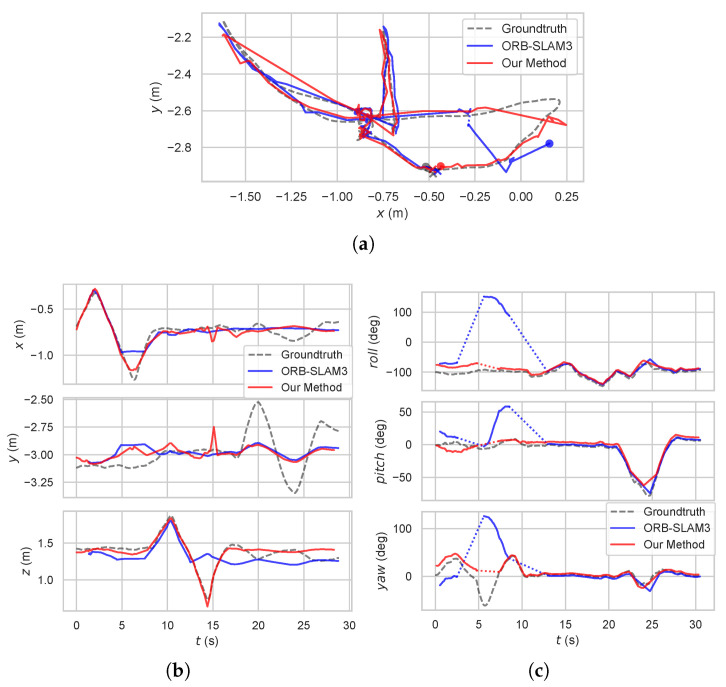
Comparison of key frame trajectories between ORB-SLAM3 and our method with ground truth in dynamic sequences: (**a**) X–Y planar trajectoryof sequence *walking_halspheres*, (**b**) translation in each direction of sequence *walking_xyz*, (**c**) rotation around each axis of sequence *walking_rpy*. Our method outperforms the baseline method in terms of the accuracy of pose estimation.

**Figure 5:**
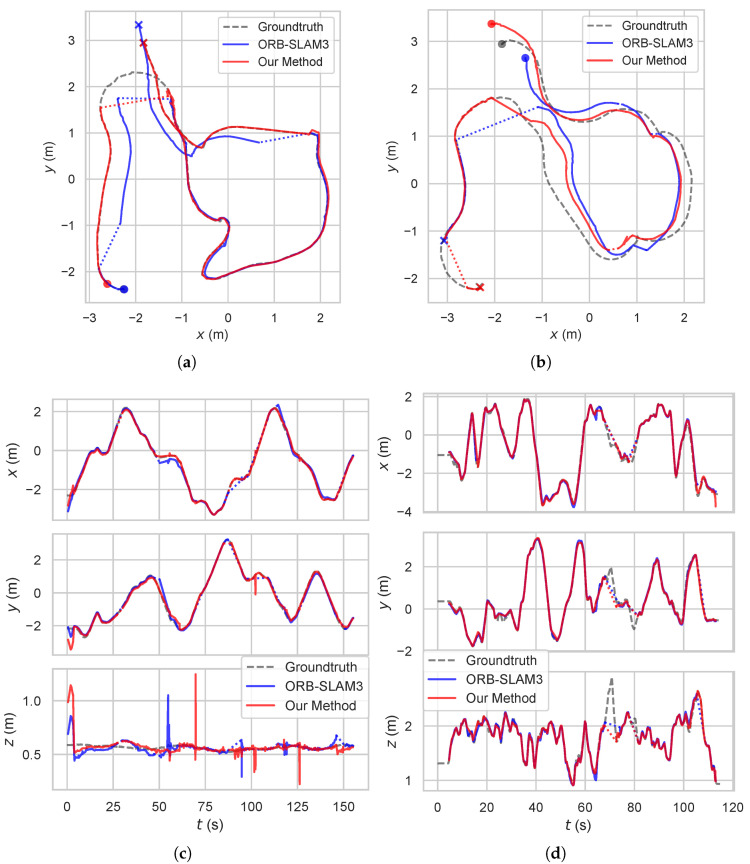
Comparison of key frame trajectories between ORB-SLAM3 and our method with ground truth in robotic sequences: (**a**) X–Y planar trajectory of sequence *pioneer_slam2*, (**b**) X–Y planar trajectory of sequence *pioneer_slam3*, (**c**) translation in each direction of sequence *pioneer_slam*, (**d**) translation in each direction of sequence *V2_03*. Our method outperforms the baseline method in terms of the completion rate of trajectory.

The metric results of the experiments on the datasets are shown in Table 2. Some of the key frame trajectories of ORB-SLAM3 and our method together with the ground truth are shown in Figure 4 and Figure 5. The colored dashed lines within the trajectory represent tracking failure.

## 7. Discussion

### 7.1. Complement of Trajectory

The overall TCR of our method surpasses the online TCR of ORB-SLAM3 across all sequences, varying from 2.23% in *V2_03* to 16.88% in *pioneer_slam*. This suggests that our method supplements lost trajectories resulting from tracking failures.

### 7.2. Reinforcement of Robustness

The online TCR of our method shows improvement compared with that of ORB-SLAM3 in many sequences, too. This indicates that the recovery of untracked frames can promote the robustness of tracking for upcoming frames.

An impressive comparison occurs in the TUM sequence *walking_halspheres*. In ORB-SLAM3, due to moving people, the system is not initialized until the 29th and 47th frame, and tracking loss occurs on the 80th frame due to the failure of local tracking; on the 139th frame, a relocalization is detected, and tracking continues till the end, achieving 91.93% online TCR. In our method, after an initialization identical to ORB-SLAM3 is performed, the 28th to the 1st frame are successfully retracked. Tracking loss does not occur afterward, indicating that the robustness of the SLAM system is promoted, achieving 97.38% online TCR and 100% overall TCR.

**Figure 6:**
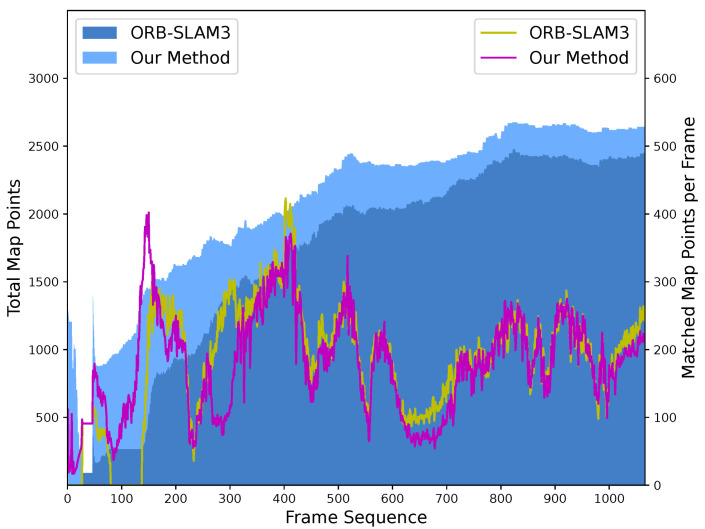
Comparison of matched map points per frame and total map points by frame between ORB-SLAM3 and our method. More map points are structured in our method, leading to an increased quantity of matched map points across most frames. Sequence: *walking_halspheres*.

**Figure 7:**
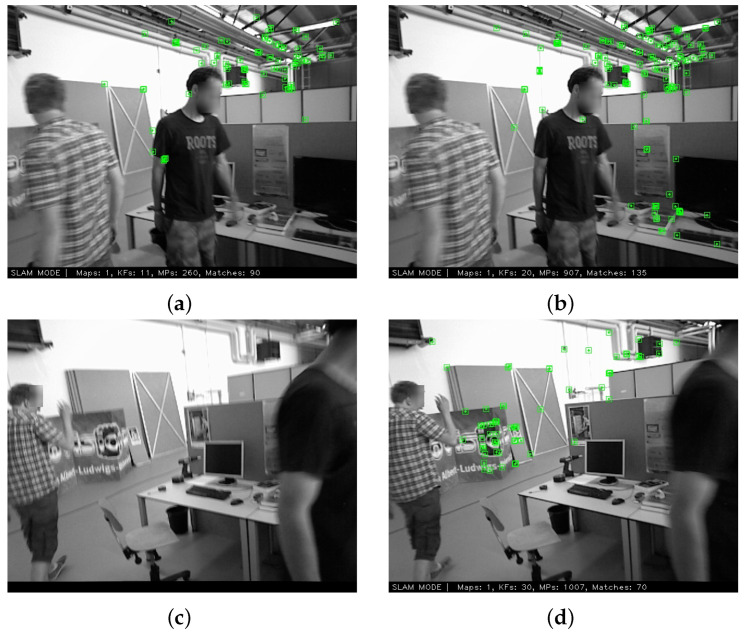
Comparison of matched map points in specific frames: (**a**) 80th frame in ORB-SLAM3, (**b**) 80th frame in our method, (**c**) 120th frame in ORB-SLAM3, (**d**) 120th frame in our method. The frames match more map points in our method than the baseline ORB-SLAM3, enhancing the robustness and accuracy of the SLAM procedure. Images are adapted from sequence *walking_halspheres* of TUM datasets.

The map points complemented by retracked frames account for this advance. The amount of matched map points per frame, together with total map points by frame, is shown in Figure 6. With the reconstruction of untracked frames, affiliated features are constructed and enrich the quantity of map points. More map points are observed by incoming frames, thereby improving the robustness of the SLAM system, especially around the 100th frame, during which tracking failure occurs in ORB-SLAM3. A comparison of the matched map points of specific frames is shown in Figure 7. In the 80th image, the frame in our method matched more map points, represented as green points, than that in ORB-SLAM3. In the 120th image, the frame in our method matched map points and remained tracked. Conversely, in ORB-SLAM3, the same image experienced tracking failure due to an insufficient number of correct matches with map points.

### 7.3. Enhancement of Accuracy

The ATE, together with RPE, is commonly lowered in our method compared with ORB-SLAM3, especially in situations of pure rotation during initialization and dynamic environments. The reduced percentage of RMS ATE varies from 0.83% in sequence *pioneer_slam* to 69.26% in sequence *pioneer_slam2*. The reduced RMS RPE can reach the highest percentage of 58.49% in the sequence *walking_halfsphere*. This suggests that the accuracy of the SLAM system can be enhanced through the reconstruction of untracked frames.

As discussed in Section 3.4, the incoming frames track more map points with complementary ones. This leads to a reduction in reprojection error and, consequently, a decrease in trajectory error. This effect is especially noticeable for preceding frames with limited matched map points.

### 7.4. On Efficiency

The reconstruction of untracked frames introduces additional processing time to the system, resulting in an increase in the mean track time.

**Figure 8:**
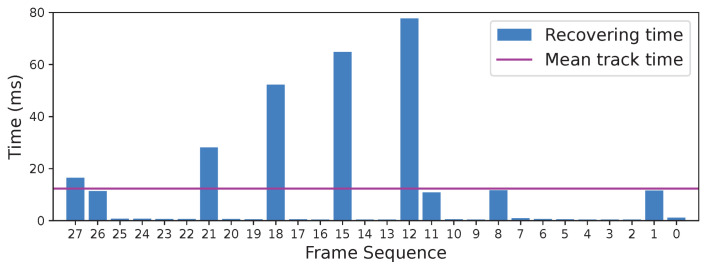
Time consumption of modeling and local BA for untracked frames and comparison with mean processing time. Frames 27, 26, 21, 18, 15, 12, 11, 8, and 1 are designated as key frames, which consume more time on bundle adjustment while retracking. The other frames are not designated as key frames and consume considerably less processing time while retracking. Sequence: *walking_halspheres*.

The time consumed by the reconstruction for untracked frames in sequence *walking_halspheres* is quantified in Figure 8. For the frames that are not designated as key frames, the processing time is 0.38 ms to 1.17 ms, significantly lower than the mean processing time, owing to the reuse of key information from elapsed images. For the frames that are designated as key frames, however, the processing time soars from 10.78 ms to 64.81 ms because of local BA, which is actually not parallelly processed in this experiment, as stated in Section 6.1.3. This makes the recovery time longer with a total delay of 295.68 ms for all 28 untracked frames, resulting in 12.09 ms of mean track time in our method compared with 11.32 ms in ORB-SLAM3. It is notable that in sequence *V2_03*, the mean track time is slightly lower in our method, a possible reason being that the map points complemented by recovered frames might decrease the tracking time further compared with ORB-SLAM3.

## 8. Conclusions and Future Works

In this paper, a method to immediately recover the poses of untracked frames for feature-based SLAM is presented. Dataset experiments conducted on the implemented system indicate that the proposed approach can not only restore the antecedent lost poses but also improve the robustness and accuracy of the tracking afterward.

A similar approach could be applied to other SLAM systems with landmark-based front-ends, where preprocessed data from untracked frames can be retrieved and reused for retracking poses and reconstructing landmarks. The proposed method may potentially enhance the performance of feature-based SLAM applications for robots and unmanned vehicles, especially in challenging environments.

As a consequence, it is essential to adapt the proposed method to actual, real-time robotic platforms to conduct lab experiments in the next step. Though the efficacy has been proved by experimental results on datasets, the additional processing time mentioned in Section 7.4 may impact performance on timeliness under these circumstances.

In further research, attention should be directed towards minimizing the latency brought by modeling extra poses and map points. One potential approach could involve adjusting the quantity of reconstructed key frames and map points to alleviate the burden of the local mapping thread, with self-adaptive parameters based on the amount of untracked frames.

## Figures and Tables

**Table 1:** Statistics on the time spending of operations in monocular ORB-SLAM3, a feature-based SLAM system. ORB extraction and pose prediction in the tracking thread account for a significant portion of the processing time.

Operations	Tracking Thread				Mapping Thread				
	**ORB Extraction**	**Pose Prediction**	**LM Track**	**KF Decision**	**KF Inserting**	**MP Culling**	**MP Creation**	**Local BA**	**KF Culling**
Time (ms)	**12.40 ± 5.10**	**1.87 ± 0.68**	4.98 ± 1.65	0.04 ± 0.03	9.25 ± 4.62	0.09 ± 0.04	22.78 ± 8.80	216.95 ± 188.77	18.88 ± 12.22
Proportion	**57.62%**	**8.69%**	23.14%	0.02%	3.40%	0.01%	8.50%	80.97%	7.05%

Data source: C Campos et al. [16].

**Table 2:** Results and comparison of dataset experiments.

Sequence		TUM						EuRoC
		**pioneer_**	**pioneer_**	**pioneer_**	**walking_**	**walking_**	**walking_**	**V2_03**
		**slam**	**slam2**	**slam3**	**xyz**	**halfsphere**	**rpy**	
Amount of Images		2921	2113	2544	859	1067	910	1922
Failure Reasons *		T, C	R, T, C	R, T, C	D	D	D, C	T, C
ORB SLAM3	Mean Track Time (ms)	**10.63**	**10.00**	**11.14**	**12.38**	**11.32**	**11.43**	13.19
	RMS ATE (cm)	19.20	20.98	40.57	25.75	9.33	8.86	15.67
	RMS RPE (cm)	**9.72**	8.23	**6.02**	15.57	7.01	5.68	59.48
	Amount of Maps	6	3	2	1	1	3	1
ORB SLAM3	Key Frames	429	288	229	75	100	105	388
	Map Points	13238	9055	7090	2369	2452	2243	14199
	Online TCR	**81.10%**	85.38%	76.92%	94.99%	91.93%	73.62%	94.02%
Our Method	Mean Track Time (ms)	16.06	11.95	15.81	14.52	12.09	20.64	**12.65**
	RMS ATE (cm)	**19.04**	**6.45**	**29.98**	**15.78**	**3.49**	**5.39**	**10.98**
	RMS RPE (cm)	10.22	**6.75**	6.09	**14.91**	**2.91**	**2.87**	**58.39**
	Amount of Maps	6	3	3	1	1	2	1
	Key Frames	716	315	350	101	106	203	401
	Map Points	19088	9758	10271	3171	2641	2738	14773
	Mean recover time (ms)	29.99	11.79	10.33	12.26	10.56	52.93	10.16
	Online TCR	79.97%	85.38%	**79.68%**	94.99%	**97.38%**	**74.83%**	**94.85%**
	Overall TCR	97.98%	93.23%	93.00%	100.00%	100.00%	87.03%	96.25%

* R: Pure rotation during monocular initialization; T: Textureless environment; D: Dynamic environment; C: Corrupt images.

## Data Availability

Data are contained within the article.

## Abbreviations

The following abbreviations are used in this manuscript:

ATE	Absolute Trajectory Error
BA	Bundle Adjustment
DLT	Direct Linear Transform
GNC	Guidance, Navigation and Control
GNSS	Global Navigation Satellite System
KF	Key Frame
KP	Key Point
LiDAR	Light Detection and Ranging
LM	Local Mapping
PnP	Perspective-n-Point
RANSAC	Random Sample Consensus
RMSE	Root Mean Square Error
RPE	Relative Pose Error
SLAM	Simultaneous Localization and Mapping
SVD	Singular Value Decomposition
TCR	Trajectory Completeness Rate
